# Predicting the daily counts of COVID-19 infection using temporal convolutional networks

**DOI:** 10.7189/jogh.13.03029

**Published:** 2023-05-26

**Authors:** Michael Li, Fatemeh Esfahani, Li Xing, Xuekui Zhang

**Affiliations:** 1 University of Victoria, Victoria, British Columbia, Canada; 2 Carnegie Mellon University, Pittsburgh, Pennsylvania, USA; 3 University of Saskatchewan, Saskatoon, Saskatchewan, Canada

The coronavirus 2019 (COVID-19) pandemic has significantly impacted the global economy and society. One of the key challenges in combating it was predicting its spread to take appropriate measures, such as lockdowns and social distancing. These measures have now been lifted, and many countries are entering the final stages of the COVID-19 pandemic.

It is essential to continue studying the data collected during the COVID-19 pandemic, even as the focus shifts to recovery and rebuilding, to improve our ability to respond to future pandemics and protect public health. The COVID-19 pandemic has provided a wealth of data that can be used to enhance our understanding of the virus and how it spreads. We used data from 3112 counties in the USA obtained from multiple sources, including the daily infection rates from the COVID-19 Data Repository of the Center for Systems Science and Engineering (CSSE) at the John Hopkins University [1], interventions used to control the spread of the virus [2], and demographics from the US Census [3], to train monitoring systems that detect and track future outbreaks or pandemics, allowing us to better prepare or even mitigate them in advance.

Artificial intelligence (AI) models have been used to forecast the cumulative daily number of COVID-19 cases. These models can analyse large amounts of data and make predictions quickly, which is critical in fast-moving pandemics. We built a forecasting model based on the temporal convolutional network (TCN) [4] and implemented a web application [5] that displays 28-day forecasts for every county in the United States. In our evaluation study, we found that our TCN-based model outperformed its extension (an ensemble model) and other state-of-art forecasting models.

## TEMPORAL CONVOLUTIONAL NETWORKS

TCNs are a deep learning method proposed by Lea et al. [4] in 2017. They are commonly used for tasks involving time-series data and can train in parallel, which results in faster training time and optimal graphical processing unit (GPU) usage. TCNs do not exhibit the vanishing gradient problem observed in recurrent neural networks [4] and can thus capture long-term dependencies in data, which is vital for accurately predicting the spread of the virus. TCNs have been used in various applications, such as flood forecasting and lip-reading recognition [6,7].

**Figure:**
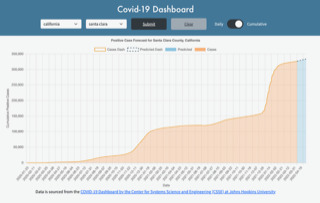
Photo: Our web application implementing methods discussed in this viewpoint, displaying 28-day forecasts for every county in the United States. Source: c0vidcather website, no permission needed for use. Available: https://www.c0vidcatcher.org/dashboard.

Our model takes a seven-day window of COVID-19 cases, which are then processed through a TCN layer of size 64. The output is then passed through a 20% dropout layer to the dense output layer, which predicts the eight day of daily cumulative COVID-19 cases (Appendix S1 in the **Online Supplementary Document**)

## ENSEMBLE MODEL

The TCN model cannot handle non-time-series data, which motivated us to extend the TCN model to an ensemble model. Our ensemble model combines multiple data sources to make predictions and uses those of different models to make a final prediction. This is advantageous, as it incorporates a broader range of variables, providing a more comprehensive overview of the situation.

Our model combines time-series data and tabular data. The time-series data consists of a seven-day window of COVID-19 cases and the tabular data contains 24 variables from the US Census used in other COVID-19 studies [1-3,8]. The tabular data are the input for a feedforward artificial neural network (ANN), while the time-series data are processed through the TCN model. The output predictions of the ANN and TCN are passed through a concatenate layer and then a dense output layer that produces the eight day's predicted daily cumulative cases. The details of our ensemble model are presented in Appendix S2 in the **Online Supplementary Document**.

## COMPARISON OF MODELS

We compared the performance of our proposed models with several state-of-the-art approaches presented in literature, including the statistical model autoregressive integrated moving average (ARIMA) [9], long short-term memory networks (LSTM) [10], convolutional neural networks (CNN), and artificial neural networks (ANN) [11].

In this evaluation study, we randomly split our data into two subsets for model training and testing. We repeated this experiment ten times to obtain confidence intervals for our comparisons and reduce the effect of the random split on our results. Each model was trained and evaluated on the same train-test split to ensure a fair comparison. As the evaluation metric, we used mean absolute errors (MAEs), which are a popular model error evaluator for forecasting continuous values. The MAE is defined as the average absolute difference between predicted and actual cases in the test data.

**Figure 1:**
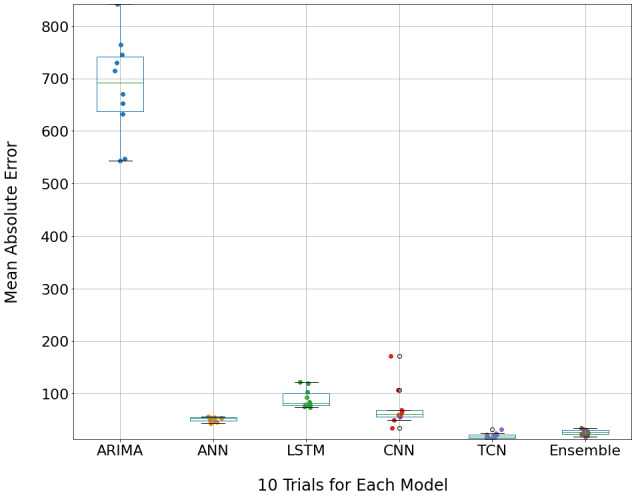
Boxplots of the MAE of six forecasting models, including the ARIMA, ANN, LSTM, CNN, TCN, and ensemble model. Each box is constructed using 10 MAEs of one method, shown as the points inside each box. The lower the box's position, the smaller the MAE values, representing the method has better prediction performance.

The MAEs of six forecasting methods over the ten random experiments are visualized as side-by-side boxplots (**Figure 1**). Smaller MAEs or lower box positions indicate better forecasting performance. We found that the TCN model outperforms all other models (mean MAE = 19.71); the ensemble model is the second best (mean MAE = 26.38), indicating the added non-temporal information cannot improve TCN’s performance. Other models had much larger mean MAEs, with ANN at 51.25, LSTM at 90.61 (LSTM), CNN at 73.51, and ARIMA at 683.86. Notably, all 10 MAE-values of TCN and ensemble model were consistently smaller than the MAEs of the four other models. Furthermore, ARIMA had a notably higher mean MAE than other models, which we believe is due to the way it trains. Since it uses a moving average estimate, any errors it has will accumulate over time. Thus, it diverges quickly over longer forecasts, an issue TCN does not have.

## CONCLUDING REMARKS

We presented the application of the TCN model for disease forecasting and demonstrated that it outperforms state-of-the-art approaches. Based on these findings, we believe that the TCN is an excellent model for forecasting during the development of pandemic monitoring systems.

Despite being the top candidate for our forecasting tool, the TCN model also has its limitations, so we suggest using it with caution. First, it requires a large amount of data to make accurate predictions, so it would not be helpful during the early stages of pandemics or new variants. However, this limitation is not unique to the TCN model and is well-known from other deep learning methods. This could potentially be solved through transfer learning and using models built for other diseases or previous variants of the same virus. Second, the TCN model can only handle time-series data. This means that it does not have a complete picture of the situation and cannot consider variables such as public transportation use and demographic characteristics. We tried using ensemble machine learning to combine the TCN model with an ANN model built from non-time-series data, but the ensemble model did not outperform the TCN model. This could be due to two reasons. First, the pattern of observed time-series data might have carried all trends encoded in the demographic variables we added, so combining them gave no new information. Second, a better ensemble method is desired to utilise information in two data sources more efficiently. Third, there are limitations with the quality of input data, since data collection may not be as accurate when positive cases increase rapidly beyond capacity. AI models and their results should be used cautiously in decision-making, and comprehensive validation is always recommended.

## Additional material


Online Supplementary Document
