# Supplementary material for: Predicting the daily counts of COVID-19 infection using temporal convolutional networks
Source: J Glob Health. 2023 May 26;13:03029. doi: 10.7189/jogh.13.03029 (PMC10208648; doi:10.7189/jogh.13.03029)
Supplement: Online Supplementary Document [file jogh-13-03029-s001.pdf]

ONLINE SUPPLEMENTARY DOCUMENT

Title

Predicting the daily counts of COVID-19 infection using temporal convolutional networks

Authors

Michael Li [1,2], Fatemeh Esfahani [1], Li Xing [3,*], Xuekui Zhang [1,*]

[1] University of Victoria, Victoria, British Columbia, Canada

[2] Carnegie Mellon University, Pittsburgh, Pennsylvania, United States

[3] University of Saskatchewan, Saskatoon, Saskatchewan, Canada

* Corresponding authors

Appendix S1: Details about our TCN model for COVID-19 case prediction

The TCN layer uses a stack of dilated convolutional layers introduced by Oord et al. [1]. The dilated convolution layers enable the neural network to look back up to (k-1)d time steps, where k is the filter size and d is the dilation factor. A neural network with a stack of multiple dilated convolutions has an increased capacity to learn long-term patterns within time-series data. Figure 1 illustrates an example of a TCN layer with an input size equal to 16. The blue, orange and white color nodes represent inputs, outputs and intermediate results, respectively. The arrows indicate the convolution of these inputs from one layer to another. In each layer, the outputs are dilated by a factor of 2.

Our input data for the TCN model is a 7-day window of COVID-19 cases, which is then followed by a TCN layer of size 64. Between each convolutional layer of the TCN model is the Rectified Linear Unit (ReLU) activation function. The output of the TCN layer is passed through a 20% dropout layer to the dense output layer, which predicts the 8th day of cumulative daily COVID-19 cases.

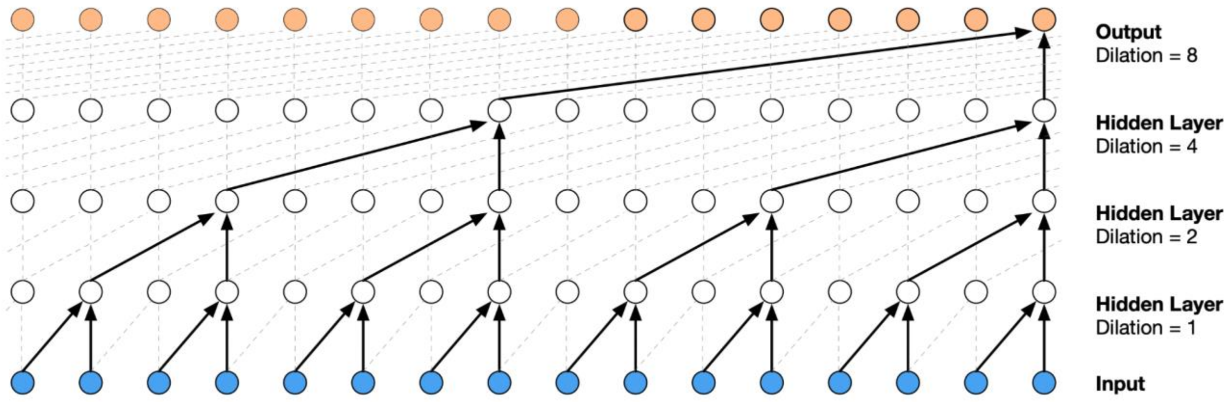

Figure S1. The architecture of TCN with dilated convolutional layers. Note: This figure is repurposed from Van den Oord et al. [1].

Appendix S2: Details about our ensemble model for COVID-19 case prediction

The ensemble model consists of two model architectures: a feedforward artificial neural network (ANN) and a TCN model. As illustrated in Figure 2, the model starts with two input branches for time series (the prior COVID-19 case counts) and tabular data (county features).

The architecture of the time-series branch for the prior COVID-19 case counts is the same as the TCN model, except that the output size is 8. The tabular data branch utilizes an ANN architecture that takes in the 24 county features and feeds them through a 20% dropout layer before applying them to a dense layer of size 8. The ReLU function is used between layers for the ANN model. The outputs of the models are combined in the concatenate layer, which appends them together to form a layer of size 16. We choose both branch output layer sizes to be 8 to treat each data type equally. A 20% dropout layer follows the concatenate layer to avoid overfitting. This is followed by a dense

output layer of size 1, which outputs our predicted 8th day of cumulative daily COVID-19 cases.

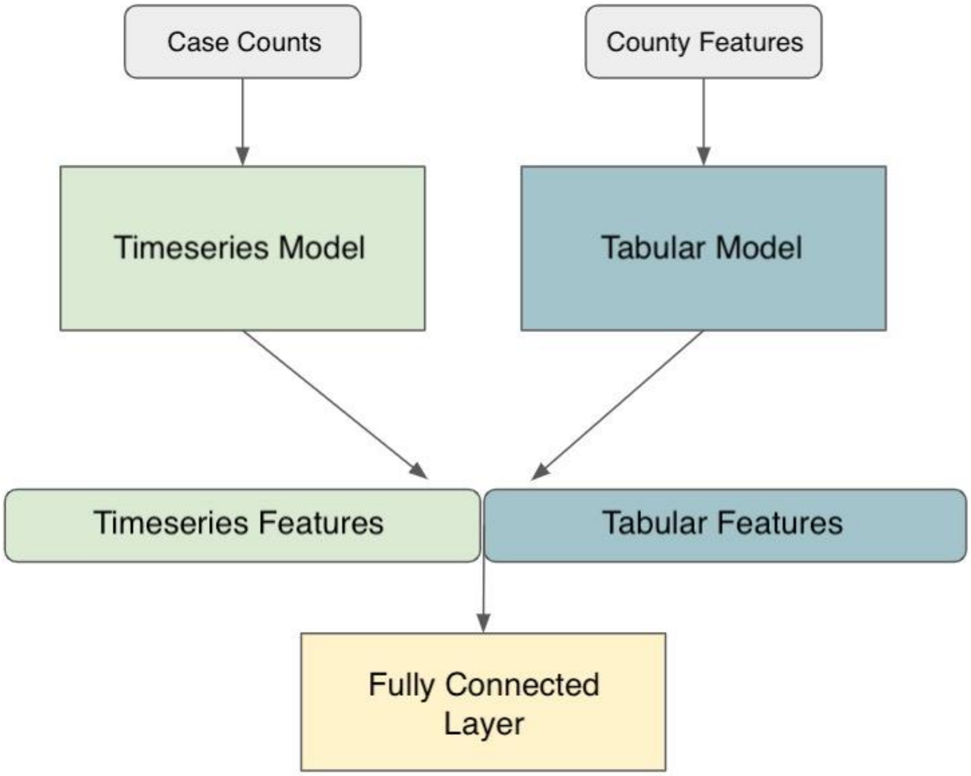

Figure S2. The architecture of the ensemble model with two branches, ANN and TCN.

REFERENCES

1 Oord AV, Dieleman S, Zen H, Simonyan K, Vinyals O, Graves A, Kalchbrenner N, Senior A, Kavukcuoglu K. Wavenet: A generative model for raw audio. arXiv preprint arXiv:1609.03499. 2016 Sep 12.

Corresponding authors:

Xuekui Zhang
University of Victoria, Victoria, BC, Canada
xuekui@uvic.ca

Li Xing
University of Saskatchewan, Saskatoon, SK, Canada
lix491@math.usask.ca
